# Association of pre-operative medication use with post-operative delirium in surgical oncology patients receiving comprehensive geriatric assessment

**DOI:** 10.1186/s12877-016-0311-5

**Published:** 2016-07-07

**Authors:** Young Mi Jeong, Eunsook Lee, Kwang-Il Kim, Jee Eun Chung, Hae In Park, Byung Koo Lee, Hye Sun Gwak

**Affiliations:** College of Pharmacy & Division of Life and Pharmaceutical Sciences, Ewha Womans University, Seoul, 03760 Republic of Korea; Department of Pharmacy, Seoul National University Bundang Hospital, Seongnam, 13620 Republic of Korea; Department of Internal Medicine, Seoul National University College of Medicine, Seoul National University Bundang Hospital, Seongnam, 13620 Republic of Korea

**Keywords:** Pre-operative medication, Post-operative delirium, Comprehensive geriatric assessment

## Abstract

**Background:**

Older patients undergoing surgery tend to have a higher frequency of delirium. Delirium is strongly associated with poor surgical outcomes. This study evaluated the association between pre-operative medication use and post-operative delirium (POD) in surgical oncology patients receiving comprehensive geriatric assessment (CGA).

**Methods:**

A total of 475 patients who were scheduled for cancer surgery and received CGA from January 2014 to June 2015 were included. Pre-operative medication review through CGA was conducted on polypharmacy (≥5 medications), delirium-inducing medications (DIMs), fall-inducing medications (FIMs), and potentially inappropriate medications (PIMs). POD was confirmed by psychiatric consultation, and DSM-V criteria were used for diagnosing delirium. The model fit of the prediction model was assessed by computing the Hosmer-Lemeshow goodness-of-fit test. Effect size was measured using the Nagelkerke R^2^. Discrimination of the model was assessed by an analysis of the area under receiver operating curve (AUROC).

**Results:**

Two models were constructed for multivariate analysis based on univariate analysis; model I included dementia and DIM in addition to age and sex, and model II included PIM instead of DIM of model I. Every one year increase of age increased the risk of POD by about 1.1-fold. DIM was a significant factor for POD after adjusting for confounders (AOR 12.78, 95 % CI 2.83-57.74). PIM was also a significant factor for POD (AOR 5.53, 95 % CI 2.03-15.05). The Hosmer-Lemeshow test results revealed good fits for both models (χ^2^ = 3.842, *p* = 0.871 for model I and χ^2^ = 8.130, *p* = 0.421 for model II). The Nagelkerke R^2^ effect size and AUROC for model I was 0.215 and 0.833, respectively. Model II had the Nagelkerke R^2^effect size of 0.174 and AUROC of 0.819.

**Conclusions:**

These results suggest that pharmacists’ comprehensive review for pre-operative medication use is critical for the post-operative outcomes like delirium in older patients.

**Electronic supplementary material:**

The online version of this article (doi:10.1186/s12877-016-0311-5) contains supplementary material, which is available to authorized users.

## Background

In Korea, the proportion of the population aged 65 years or over in 2014 was 12.7 %. By 2030, that will increase to 24.3 % and is expected to rise to 32.3 % by 2040. The most common cause of death in those ≥ 65 years of age in 2013 was cancer [1]. The proportion of older patients with cancer diagnosis and subsequent surgery is also increasing.

Older patients suffer from comorbid diseases and have a high risk of post-operative complications [2–4]. In particular, older cancer patients have a high prevalence of comorbidities (53 ~ 60 %). Among post-operative complications, older patients undergoing surgery tend to have a higher frequency of delirium [5]. Delirium is strongly associated with poor surgical outcomes. In the hospital, post-operative delirium (POD) is associated with a 2- to 5-fold increased risk of major post-operative complications, including an increased risk of death [5].

Patients on chronic drugs unrelated to their surgical procedure are more likely to have post-operative complications [6]. Older cancer patients, in particular, have a high prevalence of comorbidities (53 ~ 60 %) and take a variety of medications. It’s been reported that the proportions of polypharmacy and potentially inappropriate medication (PIM) users among older cancer patients were 84 and 51 %, respectively [7–9]. Pre-operative comprehensive geriatric assessment (CGA) is used to assess medication review in addition to comorbidities, functional status, cognitive function, nutritional status, and socioeconomic issues [10].

Pre-operative CGA can predict post-operative morbidity and mortality in older patients undergoing surgery as well as effects on the treatment plan [11, 12]. Although effectiveness of CGA as a prediction tool of adverse post-operative outcomes (i.e., identifying older patients at a greater risk of mortality, post-discharge institutionalization, adverse in-hospital events, and prolonged length of hospital stay) of older patients who are scheduled for cancer surgery in particular, has been proven, studies on the relationship between pre-operative use of medication and post-operative outcomes are rare. Considering that polypharmacy and use of psychotropic medications such as benzodiazepines, anticholinergics, antihistamines, antipsychotics are known to induce POD, pre-operative medication use should be evaluated as one of important markers to predict post-operative outcomes in CGA [13, 14]. Therefore, this study was to evaluate the association between pre-operative medication use and POD.

## Methods

### Study population and data collection

All patients ≥ 65 years of age scheduled for cancer surgery were eligible in this study. We retrospectively studied consecutive subjects ≥ 65 years of age who were scheduled for cancer surgery and presented for preoperative CGA at the geriatric center of Seoul National University Bundang Hospital between January 2014 and June 2015. We excluded patients who refused surgery or did not have cancer surgery.

Baseline patient characteristics collected from electronic medical records included age, sex, height, weight, cancer type, and comorbidities. Serum creatinine, lean body weight, sex, and age were used to estimate renal function by Cockcroft-Gault equation. The risk of delirium was measured by the Nursing Delirium Screening Scale, with scores ranging from 0 to 5; a score of 2 or higher suggests an increased risk of POD [15].

### Cognitive function evaluation and pre-operative medication review in CGA

Pre-operative CGA was performed using an established tool by the geriatric team, which consisted of geriatricians, nurse specialists, dietitians, and pharmacists. Pharmacists were wholly responsible for the drug assessment. Cognitive function was evaluated using the Korean version of the Mini-Mental Status Examination (MMSE-KC), with scores ranging from 0 to 30 (dementia is a score < 17) [16].

Medication review was performed as follows; 1) Patients were guided to carry records of their medications (prescriptions) or their actual medications in advance of CGA. 2) Pharmacists interviewed patients and caregivers to figure out all prescription and non-prescription medications. Information of PRN use was also obtained. In the process, pharmacists recorded number of medications, pre-operative discontinuation-requiring medications (PDRMs), delirium-inducing medications (DIMs), fall-inducing medications (FIMs), and PIMs.

PDRMs were defined as medications that should be discontinued before surgery due to surgical risks, such as antithrombotic agents for post-operative hemorrhage, metformin for lactic acidosis, exogenous hormones for venous thromboembolism, and herbal medications for the uncertainty over their actual contents (Table 1).

**Table 1:** Pre-operative medications reviewed for comprehensive geriatric assessment in this study

Pre-operative discontinuation-requiring medications
Aceclofenac, Anastrozole, Artemisia asiatica extract, Aspirin, Avocado-soya titrated extract, Beraprost, Celecoxib, Cilostazol, Cimicifugae rhizoma extract, Clopidogrel, Coptis rhizome extract, Dabigatran, Dalteparin, Dexibuprofen, Ginkgo biloba Leaf extract, Hedera helicis folia extract, Hypericum extract, Ibudilast, Ibuprofen, Indobufene, Clematidis Radix/Trichosanthes Root/Prunella Spike Extract, Kallidinogenase, Letrozole, Limaprost, Loxoprofen, Mefenamic acid, Meloxicam, Mesoglycan, Metformin, Milk-thistle extract, Motilitone, Nafronyl oxalate, Naproxen, Nicergoline, Pelargonium sidoides extract, Petasites hybridus folium extract, Phellinus linteus extract, Raloxifene, Rivaroxaban, Sarpogrelate, Streptokinase/Streptodornase, Sulodexide, Talniflumate, Tibolone, Triflusal, Vaccinium myrtillus extract, Vitis vinifera extract, Warfarin, Zea mays L. titrated extract, Angelica extract
Delirium-inducing medications
**Alprazolam**, Amantadine, **Amitriptylin**e, Atenolol, Benserazide/Levodopa, Bicalutamide, Bisoprolol, Buspirone, Carbamazepine, Carbidopa, Celecoxib, **Chlordiazepoxid**e, **Chlorpheniramine**, Cimetidine, Ciprofloxacin, **Clonazepam**, **Clotiazepam**, **Cyproheptadin**e, **Diazepam**, **Digoxin**, **Dimenhydrinate**, Divalproex, Donepezil, Entacapone, **Etizolam**, Famotidine, Fentanyl, **Flurazepam**, Gabapentin, Hydromorphone, **Hydroxyzine**, Ibuprofen, **Imipramine**, Lafutidine, Levofloxacin, **Lorazepam**, **Meloxicam**, Memantine, Morphine, Nizatidine, **Nortriptyline**, **Orphenadrine**, Oxycodone, Pramipexole, Prednisolone, Pregabalin, Procyclidine, Propranolol, **Quetiapine**, Ranitidine, Rivastigmine, Ropinirole, **Scopolamine**, Tiropramide, Tramadol, Trazodone, **Triazolam**, Valproate, **Zolpidem**
Fall-inducing medications
Agiocur pregranule, Acarbose, Alfuzosin, Alogliptin, Alprazolam, Amitriptyline, Amlodipine, Amosulalol, Arotinolol, Atenolol, Azelastine, Barnidipine, Benidipine, Bepotastine, Bisoprolol, Bromazepam, Calcium polycarbophil, Candesartan, Carbamazepine, Carvedilol, Cetirizine, Chlordiazepoxide/Clidinium, Chlorpheniramine, Chlorpromazine, Chlorthalidone, Cilnidipine, Clonazepam, Clotiazepam, Codeine, Desloratadine, Diazepam, Diltiazem, Dimenhydrinate, Divalproex, Doxazosin, Doxylamine, Duloxetine, Efonidipine, Enalapril, Epinastine, Eprosartan, Escitalopram, Etizolam, Felodipine, Fentanyl, Fexofenadine, Fimasartan, Flunitrazepam, Flurazepam, Furosemide, Gabapentin, Gemigliptin, Glibenclamide, Gliclazide, Glimepiride, Hydrochlorothiazide, Hydromorphone, Hydroxyzine, Hypericum extract, Imipramine, Indapamide, Insulin, Irbesartan, Isradipine, Lactulose, Lercanidipine, Levocetirizine, Levosulpiride, Linagliptin, Loratadine, Lorazepam, Losartan, Magnesium oxide, Mequitazine, Metformin, Mirtazapine, Morphine, Naftopidil, Nateglinide, Nebivolol, Nifedipine, Nortriptyline, Olmesartan, Olopatadine, Oxycodone, Paroxetine, Perindopril, Perphenazine, Pioglitazone, Piprinhydrinate, Pregabalin, Propranolol, Prucalopride, Quetiapine, Ramipril, Saxagliptin, Sertraline, Sitagliptin, Spironolactone, Telmisartan, Terazosin, Tianeptine, Torsemide, Trazodone, Triazolam, Triprolidine, Valproate, Valsartan, Venlafaxine, Verapamil, Vildagliptin, Voglibose, Zolpidem
Potentially inappropriate medications
Aceclofenac^a^, **Alprazolam**, **Amitriptyline**, Bromazepam, **Chlordiazepoxide**/clidinium, **Chlorpheniramine**, Chlorpromazine, Cimetropium, **Clonazepam**, **Clotiazepam**, **Cyproheptadine**, Desmopressin, Dexibuprofen^a^, **Diazepam**, **Digoxin**, **Dimenhydrinate**, Doxylamine, **Etizolam**, Flunitrazepam, **Flurazepam**, Glibenclamide, **Hydroxyzine**, **Imipramin**e, Levosulpiride, **Lorazepam**, Megestrol, **Meloxicam** ^a^, Metoclopramide, Naproxen^a^, **Nortriptyline**, **Orphenadrine**, Paroxetine, Perphenazine, Piprinhydrinate, **Quetiapine**, **Scopolamine**, Talniflumate^a^, Testosterone, **Triazola**m, Triprolidine, **Zolpidem**

^a^Chronic use

Drugs written in bold font are those which are included in both delirium-inducing and potentially inappropriate medications

DIMs were medications whose adverse events, such as delirium, confusion or hallucination, were reported over 1 % by the drug information database Micromedex^®^. Deliriants including high dose narcotics, benzodiazepines, and anticholinergic medications were also included in DIMs (Table 1) [17]. Antihypertensive agents, diuretics, glucose lowering agents, antihistamines, laxatives, antipsychotics, neuroleptics, antidepressants, hypnotics and sedatives, and opioids were classified as FIMs (Table 1) [18]. PIMs were determined according to the 2015 Beers criteria [19]. Levosulpiride (prokinetic agents available in Korea) was included, although it is not listed in the Beers criteria, because it frequently causes drug-induced movement disorders [20] and since safer alternatives are available (Table 1) [21].

Number of medications was counted by the number of active ingredients. Multi-component digestives, antacids, multivitamins, or herbal extracts were regarded as one ingredient because they have a single effect. Topical drugs and eye drops were not included because they rarely induce systemic adverse events. Polypharmacy was defined as taking more than five medications [22, 23].

Delirium after operation was determined by psychiatric consultation, and DSM-V criteria were used for diagnosis of delirium [24].

### Statistical analyses

The Mann–Whitney-U-test was used to compare continuous variables between patients with and without POD, and the chi-square test was used for categorical variables. Multivariable logistic regression analysis was used to identify independent risk factors for POD. Factors having *p*-values < 0.05 from univariate analysis along with strong confounders of age and sex were included in the multivariate analysis. Variables were entered by stepwise selection for *p* < 0.05 and were removed for *p* > 0.1. Odds ratio (OR) and adjusted odds ratio (AOR) were calculated from univariate and multivariate analyses, respectively. *P*-values less than 0.05 were considered statistically significant.

The model fit of the prediction model was assessed by computing the Hosmer-Lemeshow goodness-of-fit test. Effect size was measured using the Nagelkerke R^2^. Discrimination of the model was further assessed by an analysis of the area under receiver operating curve (AUROC), which assesses the ability of the risk factor to predict POD. All statistical analyses were carried out using the Statistical Package for Social Sciences version 17.0 for Windows (SPSS Inc., Chicago, IL, USA).

## Results

A total of 527 patients were scheduled for cancer surgery and received CGA from January 2014 to June 2015. Fifty two patients were excluded due to refusal for surgery (*n* = 35), changed treatment plan (*n* = 13), and age < 65 years (*n* = 4). Accordingly, data from 475 cancer patients who underwent pre-operative CGA and cancer surgery were used for the analysis.

As shown in Table 2 and Additional file 1, patients’ median age was 76 years (range 65–96); 281 patients (59.2 %) were ≥ 75 years of age and 120 (25.3 %) were ≥ 80 years of age. About 55 % of the study patients were women. More than 70 % of patients had cardiovascular diseases and 17 patients (3.6 %) had dementia. More than two-thirds of patients had creatinine clearance < 50 mL/min. Patients had gastrointestinal cancer (*n* = 353), breast cancer (*n* = 93), gynecology cancer (*n* = 15), genitourinary cancer (*n* = 5), and other cancers (*n* = 9). Delirium score ≥ 2 was found in 3.2 % of patients. Around half of patients had polypharmacy (50.5 %), PDRM (57.3 %), and DIM (42.1 %). Patients on FIM and PIM comprised 76.8 % and 26.7 %, respectively.

**Table 2:** Association of pre-operative medication use with post-operative delirium

Characteristics	No (%)	Delirium No (%)		P
		Presence (*n* = 19)	Absence (*n* = 456)	
Age (years)				0.018
Median (range)	76.0 (65.0–96.0)	79.0 (71.0–89.0)	76.0 (65.0–96.0)	
Sex				0.157
Male	215 (45.3)	12 (63.2)	203 (44.5)	
Female	260 (54.7)	7 (36.8)	253 (55.5)	
BMI (kg/m^2^)^a^				0.054
Median (range)	23.6 (15.0–40.4)	21.9 (15.6–35.1)	23.7 (15.0–40.4)	
CVD presence^b^				0.609
Yes	343 (72.2)	15 (78.9)	328 (71.9)	
No	132 (27.8)	4 (21.1)	128 (28.1)	
Diabetes Presence				1.000
Yes	125 (26.3)	5 (26.3)	120 (26.3)	
No	350 (73.7)	14 (73.7)	336 (73.7)	
Dementia Presence				0.026
Yes	17 (3.6)	3 (15.8)	14 (3.1)	
No	458 (96.4)	16 (84.2)	442 (96.9)	
CrCl^a^				0.776
≥50 mL/min	96 (21.1)	3 (15.8)	93 (21.3)	
<50 mL/min	359 (78.9)	16 (84.2)	343 (78.7)	
Cancer Type				0.179
Gastrointestinal	353 (74.3)	17 (89.5)	336 (73.7)	
Others^c^	122 (25.7)	2 (10.5)	120 (26.3)	
Delirium Risk Score^d^				0.119
0–1	454 (96.8)	17 (89.5)	437 (97.1)	
≥2	15 (3.2)	2 (10.5)	13 (2.9)	
Polypharmacy				0.159
Yes	240 (50.5)	13 (68.4)	227 (49.8)	
No	235 (49.5)	6 (31.6)	229 (50.2)	
PDRM				1.000
Yes	272 (57.3)	11 (57.9)	261 (57.2)	
No	203 (42.7)	8 (42.1)	195 (42.8)	
DIM				<0.001
Yes	200 (42.1)	17 (89.5)	183 (40.1)	
No	275 (57.9)	2 (10.5)	273 (59.9)	
FIM				0.091
Yes	365 (76.8)	18 (94.7)	347 (76.1)	
No	110 (23.2)	1 (5.3)	109 (23.9)	
PIM				<0.001
Yes	127 (26.7)	12 (63.2)	115 (25.2)	
No	348 (73.3)	7 (36.8)	341 (74.8)	

*BMI* body mass index, *CVD* cardiovascular disease, *CrCl* creatinine clearance, *PDRM* pre-operative discontinuation requiring medication, *DIM* delirium-inducing medication, *FIM* fall-inducing medication, *PIM* potentially inappropriate medication

^a^There were 20 missing data for BMI and CrCl

^b^CVD included hypertension, ischemic heart disease (unstable angina, stable angina, myocardiac infarction), dyslipidemia, heart failure, atrial fibrillation, and cerebral infarction

^c^Others included 93 breast cancer, 15 gynecology cancer, 5 genitourinary cancer, and 9 other cancer patients

^d^There were 6 missing data for delirium risk score

POD was diagnosed in 19 patients (4 %). Patients who experienced POD were older than those without POD (*p* = 0.018). Among comorbidities, dementia had a significant relationship with POD; patients with POD had more than five times higher proportion of dementia presence compared to those without POD (*p* = 0.026). Low BMI was associated with POD with marginal significance (*p* = 0.054).

More than two-fold patients in the POD group took DIM and PIM before operation (*p* < 0.001). Patients in POD group took more multiple medications and FIM rather than patients in no POD group, although statistical significance was not obtained.

As presented in Table 3, age, dementia, DIM, and PIM were significant factors for POD in the univariate analysis. Since there was multicollinearity between DIM and PIM, two models were constructed for the multivariate analysis. Model I included dementia and DIM in addition to age and sex, and model II included PIM instead of DIM of model I.

**Table 3:** Univariate and multivariate regression analyses to identify predictors for post-operative delirium

Characteristics	Unadjusted OR (95 % CI)	Adjusted OR (95 % CI)	Adjusted OR (95 % CI)
		Model I	Model II
Age	1.117 (1.029–1.212)^**^	1.098 (1.005–1.199)^*^	1.098 (1.006–1.199)^*^
Male	2.137 (0.826–5.526)	2.776 (1.029–7.490)^*^	2.921 (1.056–8.081)^*^
BMI	0.868 (0.752–1.002)		
CVD	1.463 (0.477–4.493)		
Diabetes Mellitus	1.000 (0.353–2.835)		
Dementia	5.906 (1.542–22.675)^**^	2.135 (0.507–8.998)	3.860 (0.886–16.806)
CrCl < 50 mL/min	1.446 (0.413–5.069)		
GI Cancer	3.036 (0.691–13.335)		
Delirium Risk Score ≥2	3.955 (0.826–18.925)		
Polypharmacy	2.186 (0.817–5.851)		
PDRM	1.027 (0.406–2.602)		
DIM	12.680 (2.895–55.541)^***^	12.775 (2.826–57.741)^***^	
FIM	5.654 (0.746–42.843)		
PIM	5.083 (1.955–13.220)^***^		5.525 (2.028–15.054)^***^

For model I construction, age, sex, dementia, and DIM were included for analysis. For model II construction, age, sex, dementia, and PIM were included for analysis

*BMI* body mass index, *CVD* cardiovascular disease, *CrCl* creatinine clearance, *PDRM* pre-operative discontinuation requiring medication, *DIM* delirium-inducing medication, *FIM* fall-inducing medication, *PIM* potentially inappropriate medication

^*^ *P* < 0.05, ^**^ *P* < 0.01, ^***^ *P* < 0.001

Age was a significant factor for POD. For every one year increase in age, the risk of POD increased about 1.1-fold in both models. No significant association was found between dementia and POD in both models. DIM (AOR 12.78, 95 % CI 2.83-57.74) and PIM (AOR 5.53, 95 % CI 2.03-15.05) were significant factors for POD after adjusting for confounders.

**Fig. 1:**
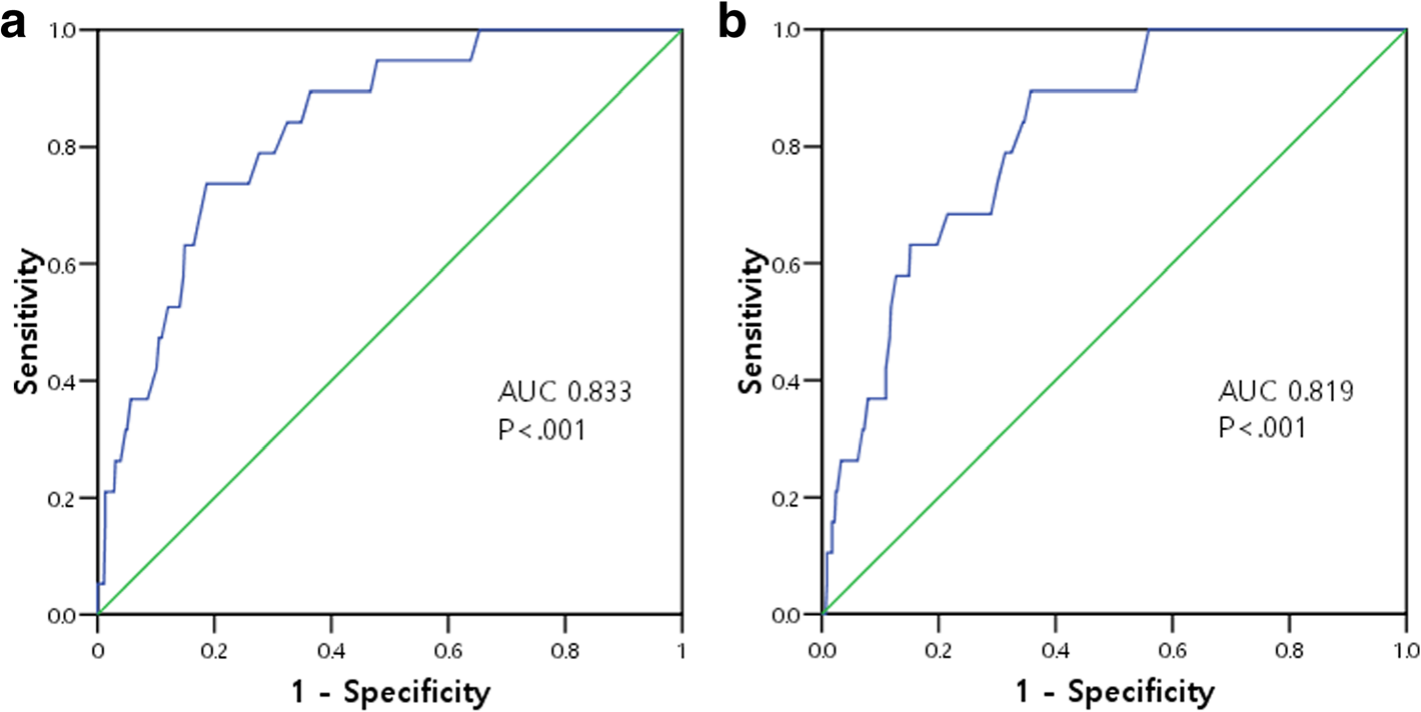
Area under receiver operating characteristic curve for post-operative delirium occurrence. **a** Model I included age, sex, and delirium-inducing medications for analysis. **b** Model II included age, sex, and potentially inappropriate medications for analysis

The Hosmer-Lemeshow test for model I, which included age, sex and DIM, revealed a good fit (χ^2^ = 3.842, *p* = .871). The Nagelkerke *R*^2^ effect size was 0.215 and AUROC was 0.833 (Fig. 1a). In model II, which included age, sex, and PIM, the Hosmer-Lemeshow test also revealed a good fit (χ^2^ = 8.130, *p* = .421), and the Nagelkerke *R*^2^effect size was 0.174. AUROC was 0.819 (Fig. 1b).

## Discussion

The main findings of this study are that one year increase in age increased risk of POD by about 1.1-fold, males had higher risk of POD (AORs of 2.8-2.9), patients with DIM had 12.8 times (95 % CI 2.8-57.7) increased risk of POD after adjusting for confounders, and patients with PIM had 5.5-fold (95 % CI 2.0-15.1) increased risk of POD after adjusting for covariates.

Consistent with our results, many studies showed that old age is significantly associated with POD [25–27]. One study reported that patients with POD were older than those without POD (median age 82 vs 74, *p* < 0.001) similar to our results (79 vs 76, *p* < 0.05) [28]. Another study on patients without dementia revealed that age was a predictor for POD (OR 1.1, 95 % CI 1.0-1.1), which is almost same as our result (AOR 1.1, 95 % CI 1.0-1.2) [29].

The association between male sex and POD is unclear. Studies on patients undergoing elective orthopedic surgery reported that male sex was a significant predictor for delirium, but only in patients without dementia [30, 31]. A systematic review of pre-operative risk factors for delirium after non-cardiac surgery also concluded that there is lack of evidence to support an association between male sex and delirium [26]. However, the study had significant heterogeneity across the study samples. Our study population was a homogeneous group with surgical oncology; around 3-fold increased risk with male sex compared to female sex was evident. One possible explanation of the higher incidence of POD in men was that men were more likely to exhibit the hyperactive form of delirium, and the incidence of hypoactive type of delirium could be underestimated due to the retrospective design of this study [32].

DIM was the strongest factor for POD in this study. More than 12-times higher risk of POD was found in pre-operative DIM users. Many studies support our results. In a study using Japanese population receiving lung and esophageal cancer surgery, use of benzodiazepines was a significant factor for POD (OR 4.0, 95 % CI 1.1-14.5) [33]. Another study also showed that significant association between pre-operative benzodiazepine use and POD (OR 3.0, 95 % CI 1.3-6.8) [34]. Around a 12-fold higher risk of POD was reported for users of propranolol, scopolamine, and flurazepam, which are included in our DIM list [35].

The American Geriatrics Society Guideline recommends PIM should be avoided in older patients due to the risk of POD [36]. Our study implicated PIM as an independent factor for POD. However, more than half drugs included in PIM list are DIM. Therefore, the effect of PIM on POD could be partially attributable to the property to induce delirium in several drugs. To estimate the effects of drugs included in both DIM and PIM, we further evaluated the association between 22 drugs included in both DIM and PIM and POD (Table 1). The OR and AOR of the 22 drugs for POD was 6.3 (95 % CI 2.4-16.1) and 8.4 (95 % CI 3.0-23.6), respectively. Therefore, drugs inducing delirium among PIM need to be more closely monitored for prevention of POD.

The model including DIM was also higher effect size (21.5 %) than that including PIM (17.4 %), although the sizes were small. However, the Hosmer-Lemeshow test results showed that the fits of both models were satisfactory. Moreover, our models had an AUROC of around 0.8, indicating that the ability of the two models to predict is much better than by chance alone (0.5). An AUROC > 0.7 for the models indicated that they had acceptable capacities for patients with POD as compared to those without POD [37].

To the best of our knowledge, this is the first study to develop prediction models by pre-operative medication use for POD and validate them using various statistical tools, such as the Hosmer-Lemeshow goodness-of-fit test, Nagelkerke R^2^, and AUROC.

However, this study has several limitations. First, the incidence of POD could be underestimated due to the definition of POD. Previous studies reported the frequency of POD in older patients as 9–29 % [25, 26]. The present incidence of 4 % was much less. Our study defined POD more strictly; POD in our study was determined with diagnosis by competent psychiatrists based on DSM-V criteria. It’s been reported that variations in definition, comorbid conditions, details related to surgery, diagnosticians, and diagnostic tools used are factors influencing incidence rates [38]. Therefore, study results could be primarily applied to patients with confirmed delirium diagnosis after cancer surgery using DSM-V by psychiatrists. Second, because of the retrospective study design, the incidence of hypoactive type of delirium might have been underestimated. Third, the adjusted odds ratio of DIM and PIM were very high because confounding effects of unmeasured comorbidities were possibly included. Therefore, our hypothesis requires further independent validation using more robust prospective designs.

In spite of the shortcomings, this study provides compelling evidence for the importance of assessing pre-operative medication. The results of this study could be utilized to develop and implement individually tailored geriatric interventions to prevent POD. Moreover, the predictive value of CGA could be greater because confirmed cases of delirium were only included in this study.

## Conclusions

The exposure to DIM and PIM in older patients before cancer surgery was associated with increased incidence of POD. This is an important finding, and should be considered in the preoperative assessment of older patients, preferably by interdisciplinary teamwork including a pharmacist.

## Abbreviation

AUROC, area under receiver operating curve; CGA, comprehensive geriatric assessment; DIM, delirium-inducing medication; FIM, fall-inducing medication; PDRM, pre-operative discontinuation requiring medication; PIM, potentially inappropriate medication; POD, post-operative delirium.

## Additional file

Additional file 1: Table S1. Raw data for the study of association between pre-operative medication use and post-operative delirium in surgical oncology patients receiving comprehensive geriatric assessment. (XLSX 129 kb)

## References

[1] Korea S (2014). Social indicator in.

[2] Turrentine FE, Wang H, Simpson VB, Jones RS (2006). Surgical risk factors, morbidity, and mortality in elderly patients. J Am Coll Surg.

[3] Deiner S, Westlake B, Dutton RP (2014). Patterns of Surgical Care and complications in Elderly Adults. J Am Geriatr Soc.

[4] Lee DH, Buth KJ, Martin BJ, Yip AM, Hirsch GM (2010). Frail patients are at increased risk for mortality and prolonged institutional care after cardiac surgery. Circulation.

[5] Marcantonio ER (2012). Postoperative delirium: a 76-year-old woman with delirium following surgery. JAMA.

[6] Kennedy JM, Van Rij AM, Spears GF, Pettigrew RA, Tucker IG (2000). Polypharmacy in a general surgical unit and consequences of drug withdrawal. Br J Clin Pharmacol.

[7] Jørgensen TL, Hallas J, Friis S, Herrstedt J (2012). Comorbidity in elderly cancer patients in relation to overall and cancer-specific mortality. Br J Cancer.

[8] Nightingale G, Hajjar E, Swartz K, Andrel-Sendecki J, Chapman A (2015). Evaluation of a pharmacist-led medication assessment used to identify prevalence of and associations with polypharmacy and potentially inappropriate medication use among ambulatory senior adults with cancer. J Clin Oncol.

[9] Maggiore RJ, Gross CP, Hurria A (2010). Polypharmacy in older adults with cancer. Oncologist.

[10] Partridge JS, Harari D, Martin FC, Dhesi JK (2014). The impact of pre-operative comprehensive geriatric assessment on postoperative outcomes in older patients undergoing scheduled surgery: A systematic review. Anaesthesia.

[11] Kim KI, Park KH, Koo KH, Han HS, Kim CH (2013). Comprehensive geriatric assessment can predict postoperative morbidity and mortality in elderly patients undergoing elective surgery. Arch Gerontol Geriatr.

[12] Badgwell B, Stanley J, Chang GJ, Katz MH, Lin HY, Ning J (2013). Comprehensive geriatric assessment of risk factors associated with adverse outcomes and resource utilization in cancer patients undergoing abdominal surgery. J Surg Oncol.

[13] Chow WB, Rosenthal RA, Merkow RP, Ko CY, Esnaola NF (2012). Optimal Preoperative Assessment of the Geriatric Surgical Patient: A Best Practices Guideline from the American College of Surgeons National Surgical Quality Improvement Program and the American Geriatrics Society. J Am Coll Surg.

[14] American Geriatrics Society Expert Panel on Postoperative Delirium in Older Adults (2015). Postoperative delirium in older adults: best practice statement from the American Geriatrics Society. J Am Coll Surg.

[15] Gaudreau JD, Gagnon P, Harel F, Tremblay A, Roy MA (2005). Fast, systematic, and continuous delirium assessment in hospitalized patients: the nursing delirium screening scale. J Pain Symptom Manage.

[16] Lee DY, Lee JH, Ju YS, Lee KU, Kim KW, Jhoo JH (2002). The prevalence of dementia in older people in an urban population of Korea: The Seoul study. J Am Geriatr Soc.

[17] Alagiakrishnan K, Wiens CA (2004). An approach to drug induced delirium in the elderly. Postgrad Med J.

[18] Woolcott JC, Richardson KJ, Wiens MO, Patel B, Marin J, Khan KM (2009). Meta-analysis of the Impact of 9 Medication Classes on Falls in Elderly Persons. Arch Intern Med.

[19] American Geriatrics Society Beers Criteria Update Expert Panel American Geriatrics Society 2015 (2015). Updated Beers Criteria for potentially inappropriate medication use in older adults. J Am Geriatr Soc.

[20] Shin HW, Kim MJ, Kim JS, Lee MC, Chung SJ (2009). Levosulpiride-induced movement disorders. Mov Disord.

[21] Reddymasu SC, Soykan I, McCallum RW (2007). Domperidone: review of pharmacology and clinical applications in gastroenterology. Am J Gastroenterol.

[22] Maher RL, Hanlon J, Hajjar ER (2014). Clinical consequences of polypharmacy in elderly. Expert Opin Drug Saf.

[23] Kaufman DW, Kelly JP, Rosenberg L, Anderson TE, Mitchell AA (2002). Recent patterns of medication use in the ambulatory adult population of the United States: the Slone survey. JAMA.

[24] American Psychiatric Association. Diagnostic and Statistical Manual of Mental Disorders. 5th ed. Arlington, VA: American Psychiateric Association Publishing; 2013.

[25] Marcantonio ER, Goldman L, Mangione CM, Ludwig LE, Muraca B, Haslauer CM (1994). A clinical prediction rule for delirium after elective noncardiac surgery. JAMA.

[26] Dasgupta M, Dumbrell AC (2006). Preoperative Risk Assessment for Delirium After Noncardiac Surgery: A Systematic Review. J Am Geriatr Soc.

[27] Raats JW, van Eijsden WA, Crolla RM, Steyerberg EW, van der Laan L (2015). Risk Factors and Outcomes for Postoperative Delirium after Major Surgery in Elderly Patients. PLoS One.

[28] Raats JW, Steunenberg SL, Crolla RM, Wijsman JH, Te Slaa A, van der Laan L (2015). Postoperative delirium in elderly after elective and acute colorectal surgery: A prospective cohort study. Int J Surg.

[29] Lee HB, Mears SC, Rosenberg PB, Leoutsakos JM, Gottschalk A, Sieber FE (2011). Predisposing factors for postoperative delirium after hip fracture repair in individuals with and without dementia. J Am Geriatr Soc.

[30] Williams-Russo P, Urquhart BL, Sharrock NE, Charlson ME (1992). Postoperative delirium: predictors and prognosis in elderly orthopedic patients. J Am Geriatr Soc.

[31] Fisher BW, Flowerdew G (1995). A simple model for predicting postoperative delirium in older patients undergoing elective orthopedic surgery. J Am Geriatr Soc.

[32] McGregor AJ, Choo EK, Becker BM, McGregor AJ, Choo EK, Becker BM (2016). Know the difference. Sex and gender in acute care medicine.

[33] Murakawa K, Kitamura Y, Watanabe S, Hongo S, Shinomiya K, Sendo T (2015). Clinical risk factors associated with postoperative delirium and evaluation of delirium management and assessment team in lung and esophageal cancer patients. J Pharm Health Care Sci.

[34] Marcantonio ER, Juarez G, Goldman L, Mangione CM, Ludwig LE, Lind L (1994). The relationship of postoperative delirium with psychoactive medications. JAMA.

[35] Rogers MP, Liang MH, Daltroy LH, Eaton H, Peteet J, Wright E (1989). Delirium after elective orthopedic surgery: risk factors and natural history. Int J Psychiatry Med.

[36] American Geriatrics Society Expert Panel on Postoperative Delirium in Older Adults (2015). American Geriatrics Society abstracted clinical practice guideline for postoperative delirium in older adults. J Am Geriatr Soc.

[37] Tangiisuran B, Scutt G, Stevenson J, Wright J, Onder G, Petrovic M (2014). Development and Validation of a Risk Model for Predicting Adverse Drug Reactions in Older People during Hospital Stay: Brighton Adverse Drug Reactions Risk (BADRI) Model. PLoS One.

[38] O’Brien D (2003). Acute postoperative delirium: definitions, incidence, recognition, and interventions. J Perianesth Nurs.

